# Antimicrobial Susceptibility Profiles of *Staphylococcus aureus* and *Streptococcus* spp. Isolates from Clinical Cases of Waterfowl in Hungary Between 2022 and 2023

**DOI:** 10.3390/antibiotics14050496

**Published:** 2025-05-12

**Authors:** Ádám Kerek, Ábel Szabó, Ákos Jerzsele

**Affiliations:** 1 Department of Pharmacology and Toxicology, University of Veterinary Medicine, István utca 2, HU-1078 Budapest, Hungary; szabo.abel@student.univet.hu (Á.S.); jerzsele.akos@univet.hu (Á.J.); 2 National Laboratory of Infectious Animal Diseases, Antimicrobial Resistance, Veterinary Public Health and Food Chain Safety, University of Veterinary Medicine, István utca 2, HU-1078 Budapest, Hungary

**Keywords:** *Staphylococcus aureus*, *Streptococcus*, antimicrobial resistance, minimum inhibitory concentration, MIC, waterfowl, geese, ducks

## Abstract

**Background**: Antimicrobial resistance (AMR) is an escalating concern in both human and veterinary medicine, particularly in the poultry sector, where antibiotic usage is substantial. *Streptococcus* spp. and *Staphylococcus aureus* are important pathogens in waterfowl, causing systemic infections. However, there is a significant lack of data regarding their antimicrobial susceptibility patterns in waterfowl populations. This study aims to address this gap by determining the minimum inhibitory concentrations (MICs) of isolates from Hungarian waterfowl farms and evaluating resistance patterns in clinical isolates. **Methods**: A total of eight *S. aureus* and 19 *Streptococcus* isolates were collected from ducks and geese between 2022 and 2023. Antimicrobial susceptibility testing was performed for 15 antimicrobials using the broth microdilution method. Potential associations between MIC values were analyzed using Spearman’s rank correlation test. **Results**: High MIC values were observed for tetracyclines, phenicols, and fluoroquinolones, in the case of *Streptococcus*, with 89.5% of isolates exhibiting resistance to doxycycline, 63.2% to florfenicol, and in the case of *S. aureus*, 25.0% to enrofloxacin. In the case of *Streptococcus*, a strong positive correlation was identified between tylosin and tiamulin (0.88, *p* < 0.001), as well as between tylosin and lincomycin (0.75, *p* < 0.001). A moderate correlation was observed between doxycycline and spectinomycin (0.72, *p* = 0.03), suggesting potential co-selection mechanisms. **Conclusions**: Our findings emphasize the necessity of continuous AMR surveillance in the waterfowl industry, particularly for multidrug-resistant strains. Understanding cross-resistance patterns is crucial for developing targeted control measures, and future studies should incorporate whole-genome sequencing to elucidate resistance determinants and co-selection mechanisms. This study highlights the potential public health and veterinary risks associated with AMR in waterfowl and reinforces the importance of responsible antibiotic use and the development of alternative therapeutic strategies in veterinary practice.

## 1. Introduction

The overuse of antimicrobial agents has significantly contributed to the spread of antimicrobial resistance (AMR). There is increasing concern that pathogenic bacteria which have developed resistance in poultry may be closely linked to the risk of human infections [1,2]. The two primary drivers of resistance development are high infection pressure on health systems and the inappropriate or inconsistent use of antibiotics [3,4]. As a result, there is growing interest in alternatives that can partially or entirely replace antibiotics, including medium-chain fatty acids [5], various plant extracts [6,7,8,9,10,11,12], antimicrobial peptides [13], and pre- and probiotics [14,15,16]. Finding alternatives to antibiotics is particularly relevant in poultry production [17], which ranks as the second largest antibiotic-consuming livestock sector after pig farming [18,19]. Given the zoonotic potential of antimicrobial-resistant bacterial strains originating from poultry, systematic surveillance is crucial to mitigate public health risks and to better understand the transmission dynamics between animal and human populations.

Waterfowl farming is a rapidly expanding industry worldwide. According to the 2020 World Waterfowl Industry Development Conference, a total of 6.44 billion ducks and 720 million geese were sold for food production in 2019 [20,21]. One of the most effective strategies for reducing antibiotic use is the implementation of biosecurity measures [22,23], alongside the application of targeted therapies based on pharmacological investigations [24,25]. In 2022, Hungary kept 2,727,000 ducks and 614,000 geese, accounting for 7.79% and 1.76% of the global duck and goose population, respectively [26].

Although poultry-related AMR research primarily focuses on broiler chickens, waterfowl remain significantly underrepresented in the literature. Ducks and geese are frequently raised in semi-intensive or open production systems with exposure to natural water sources, wild birds, and other environmental reservoirs of antimicrobial resistance. Moreover, their longer production cycles and potentially distinct antimicrobial usage patterns may contribute to unique resistance dynamics [27].

*Staphylococcus aureus* and *Streptococcus* spp. were selected for this study because they are among the most commonly isolated Gram-positive pathogens in clinical waterfowl cases, particularly in association with arthritis, septicemia, and mortality. Both genera also carry notable zoonotic potential, with *S. aureus* being a well-documented foodborne pathogen. Although other bacteria, such as *E. coli* and *Enterococcus* spp., are also relevant in the context of AMR, this study focuses on these two genera due to their economic impact, clinical prevalence, and potential public health implications [28,29,30,31].

*Staphylococcus aureus* is a widespread Gram-positive bacterium with significant pathogenic potential. In humans, it can cause a range of diseases, from skin infections to sepsis and multi-organ failure. In poultry, *S. aureus* is often a commensal microorganism but can become pathogenic under certain conditions [32,33,34,35]. Zoonotic transmission may occur through direct contact or via contaminated food products [34,36]. The emergence of methicillin-resistant *S. aureus* (MRSA) and methicillin-resistant *Staphylococcus pseudointermedius* (MRSP) is particularly concerning, as these strains pose increasing challenges in both veterinary and human medicine [35,37,38]. In recent years, *S. aureus* has also been frequently detected in poultry meat products [39,40].

Data on antimicrobial resistance in *S. aureus* strains isolated from poultry are limited. Reports from the United States indicate high levels of resistance to aminoglycosides, fluoroquinolones, macrolides, and potentiated sulfonamides, with particularly high resistance to tetracyclines [34,41]. Similar trends have been observed in Germany [42,43], Poland [44,45], Hong Kong [46], and South Korea [47,48].

The *Streptococcus* genus consists of 104 recognized species, ranging from commensals to highly virulent pathogens. These Gram-positive bacteria are widespread in the environment, including water, soil, and the surfaces, digestive tracts, and respiratory systems of animals and humans [49,50]. In human medicine, *Streptococcus pneumoniae* is a major pathogen, causing diseases from otitis media to severe systemic infections, particularly in immunocompromised individuals [50,51,52].

In waterfowl, *Streptococcus* infections most commonly occur as secondary complications of pre-existing diseases, primarily affecting young birds. These infections often result in sudden death, particularly within the first three weeks of life [53]. Due to the acute nature of the disease, clinical signs are often minimal, although neurological symptoms, such as tremors, seizures, and opisthotonus, can sometimes be observed [54]. Necropsy findings typically include hepatomegaly and a mottled spleen [55]. In ducks, the most frequent septicemic *Streptococcus* species are *Streptococcus bovis* and *Streptococcus gallolyticus* [56]. Treatment generally involves amoxicillin or oxytetracycline; however, in the long term, proper biosecurity and hygiene measures play a key role in preventing infections [22,52,55,57].

This study aligns with the overarching objectives of zoonotic disease research, emphasizing the need to monitor antimicrobial resistance in food-producing animals to assess potential spillover events. By characterizing resistance profiles in waterfowl pathogens, our findings contribute to a One Health approach aimed at mitigating zoonotic threats and transfer to humans.

The aim of this study was to provide a comprehensive analysis of the antimicrobial susceptibility profiles of *S. aureus* and *Streptococcus* spp. strains isolated from ducks and geese. Spanning multiple years, this study holds significant practical value due to the scarcity of available literature on AMR in waterfowl populations.

## 2. Results

### 2.1. Origin of the Strains

Post-mortem examinations of birds submitted to the national reference laboratory revealed symptoms of septicemia, including vascular congestion, hemorrhagic enteritis, and an enlarged, congested liver. Serofibrinous exudates were observed in the lungs, liver, and heart.

During the sample collection period, *S. aureus* was isolated in eight cases from affected joints and liver tissue, whereas *Streptococcus* spp. were identified in 19 cases from the joints, liver, lungs, and pericardium (Table 1). Of the eight *S. aureus* isolates, six originated from geese and two from ducks. Among the 19 *Streptococcus* spp. isolates, fifteen were derived from geese and four from ducks. The majority of cases (77.8%) were isolated from the liver.

### 2.2. Minimum Inhibitory Concentration Values

The *S. aureus* isolates (*n* = 8) exhibited variable susceptibility to β-lactams. Although 50% of the population displayed high sensitivity to amoxicillin (MIC_50_ = 0.5 µg/mL), the MIC value for 90% of the population was notably high (MIC_90_ = 64 µg/mL). A considerably lower MIC was observed for amoxicillin–clavulanic acid (MIC_50_ = 0.25 µg/mL, MIC_90_ = 4 µg/mL), suggesting enhanced efficacy. Ceftriaxone also demonstrated high variability, but 50% of the population showed high sensitivity (MIC_50_ = 1 µg/mL). In contrast, imipenem retained its efficacy, with an MIC_50_ value of 0.03 µg/mL and an MIC_90_ of 0.125 µg/mL.

Among protein synthesis inhibitors, the isolates exhibited high minimum inhibitory concentration (MIC) values for neomycin and spectinomycin. Amoxicillin resistance was observed in 62.5% (*n* = 5/8) of the isolates, whereas 37.5% (*n* = 3/8) remained susceptible. A similar resistance trend was noted for doxycycline. The efficacy of tylosin was 62.5% (*n* = 5/8), that of tiamulin was 25.0% (*n* = 2/8), and imipenem exhibited 100.0% (*n* = 8/8) efficacy.

For enrofloxacin, 25.0% (*n* = 2/8) of the isolates were resistant, with MIC_50_ = 1 µg/mL and MIC_90_ = 4 µg/mL. As expected, colistin displayed high MIC values, given its specificity against Gram-negative bacteria. Moderate resistance was observed for trimethoprim–sulfamethoxazole (25% resistance, *n* = 2/8), whereas 100% (*n* = 8/8) of isolates remained susceptible to vancomycin.

**Figure 1:**
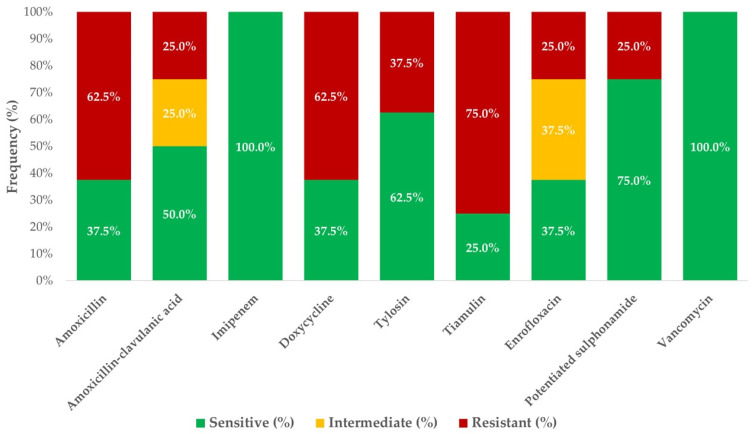
Susceptibility of *Staphylococcus aureus* (*n* = 8) strains to drugs with clinical breakpoints. The chart shows the percentage of isolates classified as susceptible (green), intermediate (yellow), or resistant (red) for each antimicrobial agent. The values inside bars represent the percentage of isolates per category. All isolates were recovered from clinical waterfowl cases between 2022 and 2023. The antibiotics for which no CLSI/EUCAST breakpoint was available are not displayed in this figure. The MIC values for these agents are provided in a Supplementary Excel File.

The susceptibility profile of the isolates, based on established clinical breakpoints, is summarized in Figure 1. The distribution of MIC values is presented in Table 2, and is presented in box plots for each antibiotic in Supplementary Figure S1. All of the MIC values are in a Supplementary Excel File.

Epidemiological cut-off values (ECOFFs) provide valuable insight into distinguishing wild-type from acquired resistance. Imipenem was the only case where the observed MIC_90_ value (0.125 µg/mL) coincided with the ECOFF, suggesting that the majority of isolates belong to the wild-type population. For amoxicillin–clavulanic acid (0.25 µg/mL), ceftriaxone (1 µg/mL), florfenicol (4 µg/mL), spectinomycin (64 µg/mL), and vancomycin (0.5 µg/mL), the MIC_50_ values were lower than the ECOFF, indicating that at least half of the isolates were part of the wild-type population. These findings highlight the importance of integrating ECOFF values with clinical breakpoints for comprehensive resistance assessments.

Among β-lactam antibiotics, 50% of the *Streptococcus* isolates exhibited high susceptibility to amoxicillin and amoxicillin–clavulanic acid (MIC_50_ = 0.5 µg/mL). However, 42.1% (*n* = 8/19) of isolates were resistant to amoxicillin–clavulanic acid, with 31.6% (*n* = 6/19) remaining susceptible and 26.3% showing intermediate susceptibility. Similar trends were observed for ceftriaxone (MIC_50_ = 0.06 µg/mL), whereas imipenem demonstrated strong efficacy (MIC_90_ = 1 µg/mL).

Regarding protein synthesis inhibitors, high MIC values were recorded for neomycin and spectinomycin. Doxycycline resistance was observed in 89.5% (*n* = 17/19) of isolates, whereas 63.2% (*n* = 12/19) of isolates were resistant to florfenicol. Elevated MIC values were also detected for tylosin, tiamulin, and lincomycin.

Enrofloxacin showed variable susceptibility patterns, with 42.1% (*n* = 8/19) of isolates susceptible, 31.6% (*n* = 6/19) exhibiting intermediate susceptibility, and 26.3% (*n* = 5/19) classified as resistant. As expected, colistin exhibited high MIC values, reinforcing its inefficacy against Gram-positive bacteria. Trimethoprim–sulfamethoxazole displayed limited efficacy, with only 15.8% (*n* = 3/19) of isolates susceptible, 31.6% (*n* = 6/19) exhibiting intermediate resistance, and 52.6% (*n* = 10/19) classified as resistant. Vancomycin susceptibility was maintained in 94.7% (*n* = 18/19) of isolates, whereas 5.3% (*n* = 1/19) displayed resistance.

**Figure 2:**
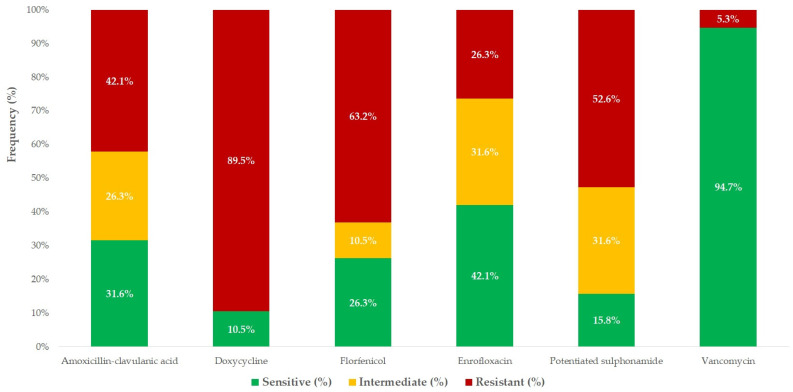
Susceptibility of *Streptococcus strains* (*n* = 19) to drugs with clinical breakpoints. The chart illustrates the percentage of isolates classified as susceptible (green), intermediate (yellow), or resistant (red) for each antimicrobial agent, based on minimum inhibitory concentration (MIC) values. The values inside each bar represent the proportion of isolates (%) in each susceptibility category. All isolates were derived from clinical samples obtained from waterfowl between 2022 and 2023. The antibiotics for which no CLSI/EUCAST breakpoint was available are not displayed in this figure. The MIC values for these agents are provided in a Supplementary Excel File.

The susceptibility profile of *Streptococcus* isolates, based on clinical breakpoints, is depicted in Figure 2, and the corresponding MIC distributions are summarized in Table 3 and presented in box plots for each antibiotic in Supplementary Figure S2. All of the MIC values are in a Supplementary Excel File.

Colistin was included as part of the standard testing panel, despite its lack of clinical application in Gram-positive infections. The high MIC values observed are in line with the known intrinsic resistance of *S. aureus* and *Streptococcus* spp.

Vancomycin was the only case where the MIC_90_ value (0.5 µg/mL) was below the ECOFF, suggesting that the majority of isolates belong to the wild-type population. For enrofloxacin (1 µg/mL), the MIC_50_ value was lower than the ECOFF, indicating that at least half of the isolates could be classified as part of the wild-type population.

**Figure 3:**
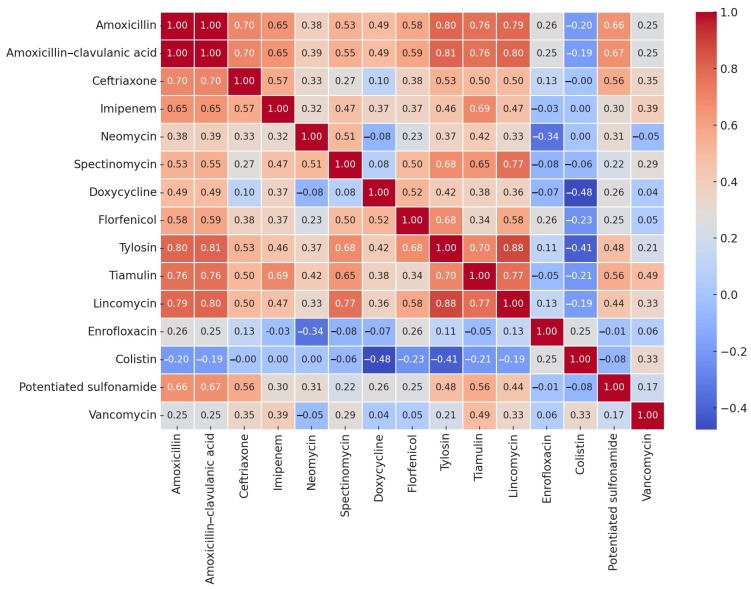
Correlation analysis of minimum inhibitory concentration (MIC) values for *Streptococcus* strains (*n* = 19) by active substance. The heatmap shows pairwise Pearson correlation coefficients between the MIC values of 15 antibiotics tested across all isolates. The warmer colors (red) indicate stronger positive correlations (r > 0), suggesting potential co-resistance patterns or shared resistance mechanisms. The cooler colors (blue) indicate negative correlations (r < 0), and white indicates weak or no correlation. The values in each cell represent the exact correlation coefficient (r) between the respective antibiotic pairs.

Given the sample size for *Streptococcus* spp. (*n* = 19), a Spearman correlation analysis was performed, as MIC values typically follow a non-normal distribution. This allowed for the assessment of both linear and monotonic relationships, which are crucial in evaluating cross-resistance potential. The correlation results are visualized in a heatmap (Figure 3).

A strong correlation was observed between amoxicillin and amoxicillin–clavulanic acid (1.00), as well as between tylosin and lincomycin (0.88). Lincomycin was strongly correlated with tiamulin and tylosin, as well as amoxicillin (0.79) and amoxicillin–clavulanic acid (0.80). Conversely, colistin exhibited weak negative correlations with most other antibiotics but a strong negative correlation with doxycycline (−0.48) and with tylosin (−0.41).

## 3. Discussion

The present study revealed high rates of AMR in *S. aureus* and *Streptococcus* spp. isolates from ducks and geese in Hungary, with particularly elevated resistance observed against β-lactams, macrolides, aminoglycosides, and doxycycline. These findings underscore the emerging threat of multidrug-resistant (MDR) Gram-positive pathogens in waterfowl production systems.

AMR is a major global challenge that requires a multidisciplinary approach integrating public health, animal health, and environmental sciences. The poultry industry, a cornerstone of global food security, faces substantial challenges due to the widespread use of antibiotics, which has driven the selection and dissemination of MDR bacteria [58,59,60]. These resistant bacteria can be transmitted to humans via multiple pathways, including through the food chain, direct contact with animals, and environmental exposure [61].

Given the intersection between public and animal health, integrated approaches, such as the One Health framework, are urgently needed. In Hungary, the veterinary antimicrobial consumption rate is 69.9 mg/PCU, positioning the country in the mid-range among European nations. Although Hungary’s total antibiotic sales (120 tons) may seem relatively low compared to Spain (1027.2 tons) or Italy (585.4 tons), the proportional use in livestock sectors remains a significant concern, particularly in poultry production [62,63].

Most poultry-related AMR research (85–90%) focuses on broiler chickens, leaving the waterfowl sector significantly underrepresented in scientific literature. This disparity reflects the economic and nutritional significance of chickens, which places them at the forefront of AMR research priorities [58]. Our study addresses this knowledge gap by characterizing AMR profiles in waterfowl pathogens, providing novel insights into *S. aureus* and *Streptococcus* spp. infections in ducks and geese.

*S. aureus* and *Streptococcus* spp. negatively impact poultry production, causing significant economic losses through arthritis, tenosynovitis, osteomyelitis, pododermatitis, and femoral head necrosis, which ultimately lead to lameness and compromised flock performance. Despite ongoing efforts, antibiotic treatment of such infections remains a major challenge, particularly in light of the high resistance rates observed in this study [64,65].

Our *S. aureus* isolates showed resistance rates of 62.5% (*n* = 5/8) to amoxicillin, 25% (*n* = 3/8) to enrofloxacin, and elevated MICs to neomycin and spectinomycin, indicating substantial therapeutic limitations. In some developing countries, misuse of antibiotics in poultry production has led to an increase in MDR *Staphylococcus* strains, defined as strains that are resistant to at least three classes of antibiotics, posing an escalating public health risk [66,67]. In a study by Eid et al. (2019), no enrofloxacin resistance was observed in *S. aureus* isolates, whereas neomycin resistance was reported at 20%, potentiated sulfonamide resistance at 80%, spectinomycin resistance at 90%, and universal resistance to amoxicillin [68]. In contrast, our findings demonstrated 25% (*n* = 2/8) enrofloxacin resistance, an MIC_50_ of 32 µg/mL for neomycin, 25% (*n* = 2/8) resistance to trimethoprim–sulfamethoxazole, an MIC_50_ of 64 µg/mL for spectinomycin, and 62.5% (*n* = 5/8) resistance to amoxicillin. Compared to these data, our findings suggest regional variation in resistance profiles, with lower sulfonamide resistance but higher enrofloxacin resistance.

Over recent years, the selective pressure exerted by antibiotics in poultry production has accelerated the spread of resistant bacteria [4,69,70]. Our results confirmed high levels of resistance to amoxicillin and doxycycline, whereas no vancomycin-resistant *S. aureus* strains were detected. Fortunately, vancomycin-resistant *S. aureus* strains remain relatively rare [66].

Similarly, *Streptococcus* spp. isolates exhibited significant resistance to β-lactams, macrolides, doxycycline, and lincosamides. However, some antibiotics, such as vancomycin, remained effective, whereas others, such as doxycycline, displayed high resistance rates. These findings emphasize the need for continuous AMR surveillance and the identification of alternative therapeutic options in the waterfowl sector.

The correlation analysis revealed a strong association between amoxicillin and amoxicillin–clavulanic acid (1.00), which is expected given their shared β-lactam structure. Notably, β-lactamase production is not a common resistance mechanism in *Streptococcus* spp. [51]. A strong correlation was also observed between tylosin and tiamulin (0.88), as well as lincomycin with tiamulin and tylosin, suggesting the presence of macrolide–lincosamide–streptogramin (MLS) cross-resistance mechanisms. This ribosomal target modification-based resistance is typically plasmid- or transposon-mediated [71]. Additionally, a significant positive correlation was detected between doxycycline and spectinomycin (0.72). Although these antibiotics inhibit protein synthesis via different mechanisms (doxycycline: ribosomal binding, spectinomycin: elongation factor inhibition), their co-occurrence suggests potential co-selection mechanisms, possibly linked to plasmid- or transposon-associated resistance determinants, such as Tn1545 and Tn917, which are known to carry *tet* and *erm* genes [72]. In contrast, colistin displayed weak correlations with other antibiotics, reinforcing its inefficacy against Gram-positive bacteria [73].

The zoonotic potential of *S. aureus* and *Streptococcus* spp. further elevates the public health relevance of our findings. Potential transmission routes from waterfowl to humans include direct contact, foodborne exposure, and environmental contamination, emphasizing the need for stringent biosecurity protocols and antimicrobial stewardship.

This study has several limitations. First, the number of isolates was relatively limited, which may restrict the generalizability of the findings. Second, molecular analyses of resistance determinants (e.g., *mecA*, *erm*, *tet*, or integron elements) were not performed, which limits mechanistic interpretation. Third, the isolates were obtained from clinical submissions, not from a systematic epidemiological sampling scheme. Despite these constraints, this study provides important baseline data for AMR monitoring in waterfowl and highlights key areas for future research.

The findings of this antimicrobial susceptibility study highlight significant antibiotic resistance patterns among *Streptococcus* and *S. aureus* isolates from waterfowl. The correlation analysis of MIC values identified strong associations between certain antibiotics, suggesting the potential presence of cross-resistance or co-selection mechanisms.

These results underscore the critical need for ongoing antimicrobial resistance monitoring in the waterfowl industry, particularly for the detection of multidrug-resistant strains. Based on the observed resistance patterns, further genetic investigations, such as the molecular detection of resistance genes, are recommended to enhance our understanding of resistance mechanisms and transmission pathways.

The increasing trend of resistant pathogenic bacteria in waterfowl raises significant public health and veterinary concerns, emphasizing the need for responsible antibiotic use strategies and the development of alternative therapeutic approaches in veterinary practice.

## 4. Materials and Methods

### 4.1. Origin of Strains

Sample collection was conducted between 2022 and 2023. The isolates were obtained from clinical cases, with isolation and pure culture preparation performed by the National Food Chain Safety Office, Directorate of Veterinary Diagnostics. Bacterial isolation was conducted using standard aerobic culture techniques on blood agar and selective media from tissue samples collected during necropsy (brain ventricle, joint, liver, pericardium, and lung). Preliminary identification was based on colony morphology and hemolysis patterns, followed by confirmation using standard biochemical tests. The isolates originated from 27 clinical cases submitted to the National Reference Laboratory between 2022 and 2023, including 21 geese and 6 ducks. Sampling was targeted, as all samples were obtained from animals with diagnosed bacterial infections. All animals were from commercial waterfowl farms. The geographical origins of the samples were classified into Hungary’s seven administrative regions: Közép-Magyarország, Közép-Dunántúl, Nyugat-Dunántúl, Dél-Dunántúl, Észak-Magyarország, Észak-Alföld, and Dél-Alföld. The pure cultures were then provided to our research group based on a mutual agreement. The isolates received on Petri dishes were stored in a Microbank™ system (Pro-Lab Diagnostics, Richmond Hill, ON, Canada) at −80°C until further analysis.

Each sample was assigned a unique identifier, and relevant metadata, including the host species (duck or goose), the affected organ (brain ventricle, joint, liver, pericardium, and lung), and geographical origin, were recorded. The geographical locations were categorized into Hungary’s seven administrative regions for systematic data management.

### 4.2. Preparation of Antibiotic Stock Solutions

The preparation of antibiotic stock solutions was conducted according to Clinical and Laboratory Standards Institute (CLSI) guidelines [74,75]

Amoxicillin and amoxicillin–clavulanic acid (2:1 ratio) were dissolved in phosphate-buffered solution (pH 7.2, 0.01 mol/L). Imipenem was prepared in phosphate-buffered solution (pH 6, 0.1 mol/L). Ceftriaxone, doxycycline, spectinomycin, neomycin, colistin, tiamulin, tylosin, lincomycin, and vancomycin were dissolved in distilled water. Trimethoprim–sulfamethoxazole (in a 19:1 ratio) was prepared by dissolving sulfamethoxazole in hot water with a few drops of 2.5 mol/L NaOH, whereas trimethoprim was dissolved in 0.05 mol/L HCl in distilled water. Enrofloxacin was dissolved in distilled water with a few drops of 1 mol/L NaOH. Florfenicol was prepared using a few drops of 95% ethanol and distilled water.

For each antibiotic, a 1024 µg/mL stock solution was prepared and was adjusted for purity according to the manufacturer’s specifications (Merck KGaA, Darmstadt, Germany).

### 4.3. Determination of Minimum Inhibitory Concentration

The phenotypic expression of AMR was assessed by determining the MIC of each isolate. This study was performed following CLSI methodology [75] and breakpoints were determined based on CLSI guidelines [74].

Bacterial isolates stored at −80 °C were cultured overnight in 3 mL Mueller–Hinton broth (MHB) at 37 °C for 18–24 h prior to testing. MIC determinations were carried out in 96-well microtiter plates (VWR International, LLC, Debrecen, Hungary). All wells, except those in the first column, were pre-filled with 90 µL of MHB. Two-fold serial dilutions of antibiotic stock solutions were prepared in MHB, starting at 512 µg/mL, covering a concentration range of 512 to 0.0009 µg/mL.

A 180 µL aliquot of the prepared antibiotic dilutions was transferred into the first column of the test plate, followed by two-fold serial dilutions across subsequent columns. After preparing the dilution series, 10 µL of a 0.5 McFarland standard bacterial suspension (105 CFU/mL) was inoculated into wells from column 11 backward using a nephelometer (ThermoFisher Scientific, Budapest, Hungary) [75]. Column 11 was the positive control (containing only bacterial suspension and broth). Column 12 was the negative control (containing only broth).

The plates were incubated at 41 °C for 18–24 h, and the MIC values were determined using the Sensititre™ SWIN™ automatic MIC reader (ThermoFisher Scientific, Budapest, Hungary) and VIZION™ system software (v3.4, ThermoFisher Scientific, Budapest, Hungary, 2024). The quality control strains used were *S. aureus* (ATCC 12600) and *Streptococcus suis* (ATCC 43765).

### 4.4. Statistical Analysis

Statistical analyses were performed using R (v4.2.2) in the RStudio environment [76]. Descriptive statistics and correlation analyses were applied to evaluate antimicrobial susceptibility data. The MIC values were log-transformed for visualization, and Spearman’s rank correlation test was conducted to assess potential relationships between the MIC values [77].

Spearman’s correlation coefficient was calculated for each pair of antibiotics. This non-parametric test is appropriate for detecting monotonic relationships, regardless of normality assumptions. Statistical significance was determined using *p*-values (<0.05), indicating significant correlations.

Results were visualized using heatmaps, allowing the identification of potential cross-resistance and co-selection patterns among antimicrobial agents.

## Figures and Tables

**Table 1:** Distribution of organ localization of isolated bacterial strains.

Bacterial Species	Pericardium	Lungs	Liver	Articulation
	*n* (%)			
*Staphylococcus aureus* (*n* = 8)	0 (0.0)	0 (0.0)	6 (75.0)	2 (25.0)
*Streptococcus* spp. (*n* = 19)	2 (10.5)	1 (5.3)	15 (78.9)	1 (5.3)
Total (*n* = 27)	2 (7.4)	1 (3.7)	21 (77.8)	3 (11.1)

**Table 2:** Distribution of the minimum inhibitory concentrations (MICs) of *Staphylococcus aureus* strains isolated from waterfowl (*n* = 8) and the calculated values for 50% (MIC_50_) and 90% (MIC_90_) of the population. The vertical red lines indicate clinical breakpoints. The vertical green lines indicate the threshold distinguishing wild-type from non-wild-type strains.

Antibiotics	Breakpoint ^1^ (μg/mL)	Distribution of Strains by MIC (μg/mL)																	MIC_50_	MIC_90_	ECOFF ^4^
		0.015	0.03	0.06	0.125	0.25	0.5	1	2	4	8	16	32	64	128	256	512	1024	µg/mL		
Amoxicillin	0.5			2	0	1	2	0	0	1	0	0	0	1	0	0	1		0.5	64	0.5
Amoxicillin–clavulanic acid ^2^	1				2	1	2	0	0	2	0	0	0	1					0.25	4	0.5
Ceftriaxone	-				3	0	0	1	0	1	1	1	0	0	0	0	1		1	16	8
Colistin	-											1	4	0	0	0	1	2	32	1024	-
Doxycycline	0.5				3	0	0	0	2	0	2	0	1						2	8	0.5
Enrofloxacin	4				1	0	2	2	1	1	0	0	0	0	1				1	4	-
Florfenicol	-							2	1	1	2	1	0	0	0	0	1		4	16	8
Imipenem	8	1	3	2	1	0	0	0	1										0.03	0.125	0.125
Lincomycin	-							2	1	1	0	0	0	1	1	0	1	1	4	512	2
Neomycin	-											1	3	2	0	1	1		32	256	1
Trimethoprim–sulfamethoxazole ^3^	4							4	2	0	0	0	1	1					1	32	0.25
Spectinomycin	-												1	4	0	1	1	1	64	512	128
Tiamulin	4						1	1	0	2	0	1	0	1	1	0	0	1	4	128	2
Tilozin	64				3	0	0	0	1	1	0	0	0	1	1	0	0	1	2	128	-
Vancomycin	32						4	1	1	2									0.5	4	2

^1^ Clinical Laboratory Standard Institute (CLSI); ^2^ 2:1 ratio; ^3^ 1:19 ratio; ^4^ European Committee on Antimicrobial Susceptibility Testing—Epidemiological cut-off value; MIC_50_—Minimum Inhibitory Concentration at which 50% of isolates are inhibited; MIC_90_—Minimum Inhibitory Concentration at which 90% of isolates are inhibited; ECOFF—Epidemiological Cut-Off Value separating wild-type (non-resistant) populations from those with acquired resistance.

**Table 3:** Distribution of the minimum inhibitory concentrations (MICs) of *Streptococcus* strains isolated from waterfowl (*n* = 19) and the calculated values for 50% (MIC_50_) and 90% (MIC_90_) of the population. The vertical red lines indicate clinical breakpoints. The vertical green lines indicate the threshold distinguishing wild-type from non-wild-type strains.

Antibiotics	Breakpoint ^1^ (μg/mL)	Distribution of Strains by MIC (μg/mL)																	MIC_50_	MIC_90_	ECOFF ^4^
		0.015	0.03	0.06	0.125	0.25	0.5	1	2	4	8	16	32	64	128	256	512	1024	µg/mL		
Amoxicillin	-		1	2	3	0	5	3	2	0	3								0.5	8	0.06 ^5^
Amoxicillin–clavulanic acid ^2^	1		1	2	3	0	5	2	0	0	6								0.5	8	0.06 ^5^
Ceftriaxone	-		4	6	2	0	1	1	1	3	0	0	1						0.06	4	0.06 ^6^
Colistin	-							1	1	0	0	8	6	0	2	0	0	1	16	128	-
Doxycycline	0.5		1	0	1	0	0	0	0	3	0	6	7	0	1				16	32	0.5 ^7^
Enrofloxacin	4					2	6	4	2	2	1	1	1						1	16	2 ^8^
Florfenicol	8						1	0	4	2	5	0	1	1	2	1	2		8	512	4 ^7^
Imipenem	-		8	3	4	0	0	3	1										0.06	1	-
Lincomycin	-				1	0	0	1	2	0	1	1	1	1	3	0	3	5	128	1024	1 ^9^
Neomycin	-													14	1	1	3		64	512	-
Trimethoprim–sulfamethoxazole ^3^	4				2	0	1	1	5	2	3	0	0	5					4	64	0.25 ^7^
Spectinomycin	-													10	2	3	1	3	64	1024	-
Tiamulin	-					1	0	0	0	0	0	1	3	2	8	1	0	3	128	1024	-
Tilozin	-				4	0	1	0	0	1	1	1	1	1	2	1	3	3	64	1024	1 ^8^
Vancomycin	2				2	3	13	0	0	1									0.5	0.5	1 ^6^

^1^ Clinical Laboratory Standard Institute (CLSI); ^2^ 2:1 ratio; ^3^ 1:19 ratio; ^4^ European Committee on Antimicrobial Susceptibility Testing—Epidemiological cut-off value; ^5^ *Streptococcus equi*; ^6^ *Streptococcus pneumonia*; ^7^ *Streptococcus suis*; ^8^ *Streptococcus dysgalactiae*; ^9^ *Streptococcus canis*; MIC_50_—Minimum Inhibitory Concentration at which 50% of isolates are inhibited; MIC_90_—Minimum Inhibitory Concentration at which 90% of isolates are inhibited; ECOFF—Epidemiological Cut-Off Value separating wild-type (non-resistant) populations from those with acquired resistance.

## Data Availability

The data presented in this study are available from the corresponding author upon reasonable request.

## Supplementary Materials

The following supporting information can be downloaded at: https://www.mdpi.com/article/10.3390/antibiotics14050496/s1, Figure S1: The distribution of minimum inhibitory concentration (MIC) values of *Staphylococcus aureus* isolates (*n* = 8) from waterfowl, visualized using a box plot for each antibiotic. Figure S2: The distribution of minimum inhibitory concentration (MIC) values of *Streptococcus isolates* (*n* = 8) from waterfowl, visualized using a box plot for each antibiotic. Supplementary Excel.

## Abbreviations

The following abbreviations are used in this manuscript:

AMR	Antimicrobial resistance
CLSI	Clinical and Laboratory Standards Institute
ECOFF	Epidemiological cut-off values
MHB	Mueller–Hinton broth
MIC	Minimum inhibitory concentration
