# Supplementary material for: Antimicrobial Susceptibility Profiles of Staphylococcus aureus and Streptococcus spp. Isolates from Clinical Cases of Waterfowl in Hungary Between 2022 and 2023
Source: Antibiotics (Basel). 2025 May 12;14(5):496. doi: 10.3390/antibiotics14050496 (PMC12108361; doi:10.3390/antibiotics14050496)
Supplement: Supplementary file 1 [file antibiotics-14-00496-s001.zip › Supplementary materials.pdf]

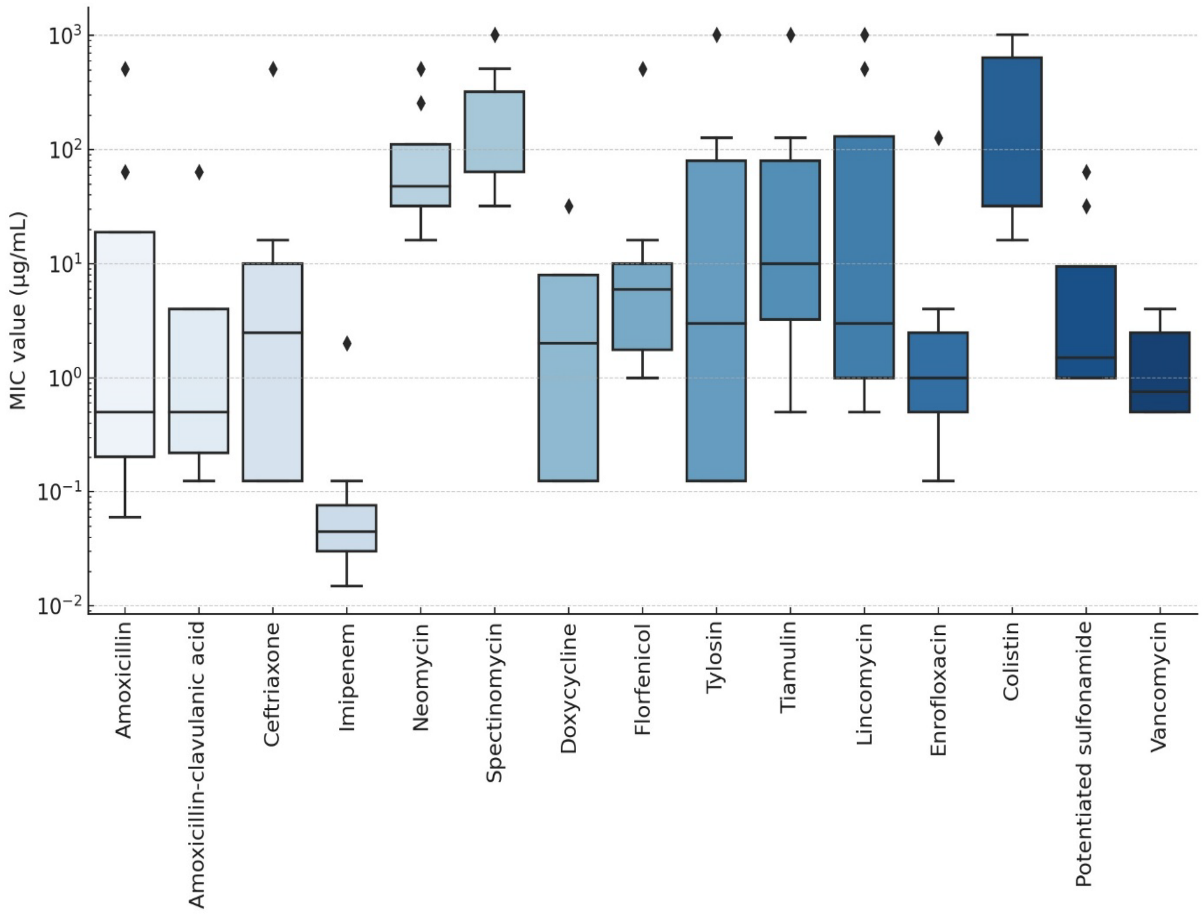

Supplementary Figure S1 The distribution of minimum inhibitory concentration (MIC) values of *Staphylococcus aureus* isolates (*n*=8) from waterfowl, visualized using a box plot for each antibiotic.

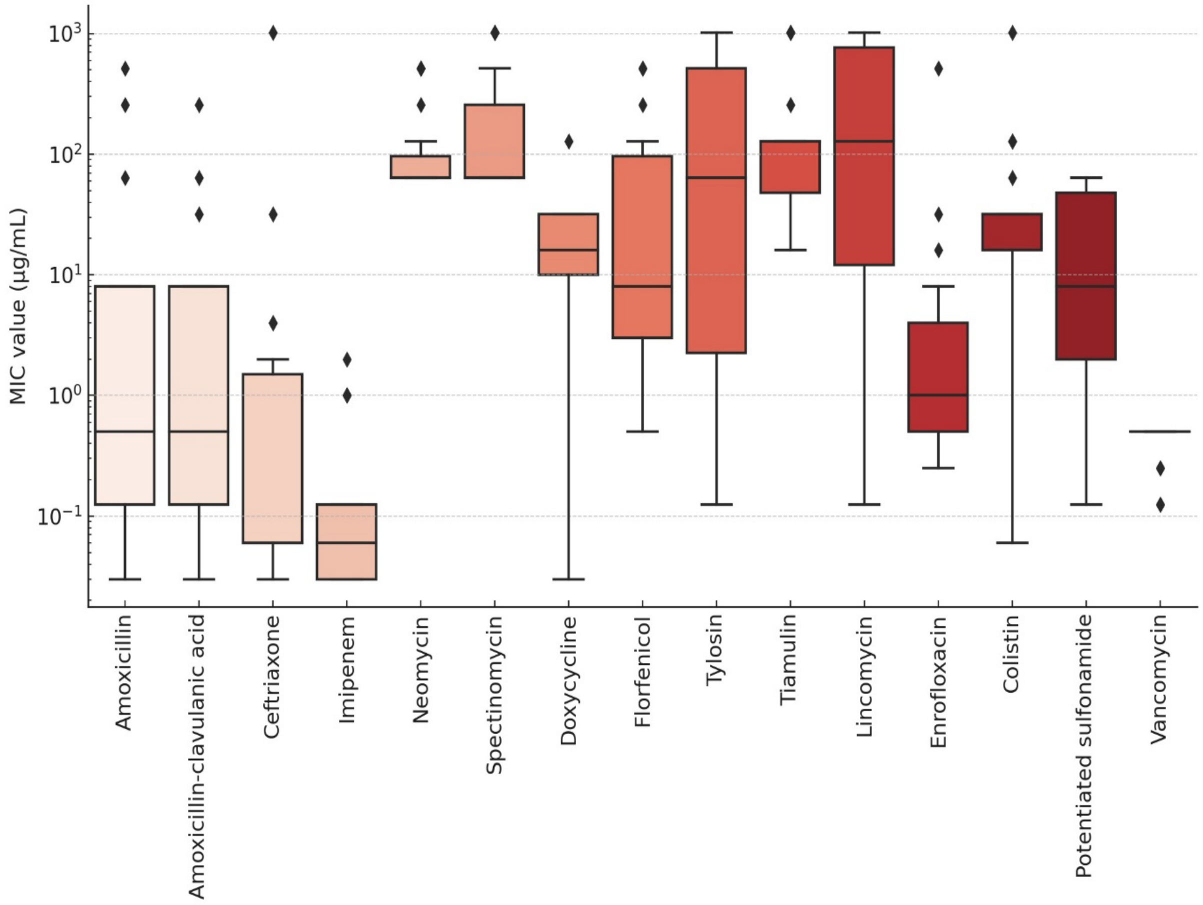

Supplementary Figure S2 The distribution of minimum inhibitory concentration (MIC) values of *Streptococcus isolates* (*n*=8) from waterfowl, visualized using a box plot for each antibiotic.
